# Alterations of circulating exosomal microRNAs in an LPS‐induced depression model of male mice: Potential role in the anti‐depressive effects of acupuncture

**DOI:** 10.14814/phy2.70310

**Published:** 2025-04-07

**Authors:** Jade Heejae Ko, Ka Yoon Chun, Seung‐Nam Kim

**Affiliations:** ^1^ College of Korean Medicine Dongguk University Goyang Korea; ^2^ Department of Neurosurgery Stanford University School of Medicine Stanford California USA

**Keywords:** acupuncture, bioinformatics, biomarkers, depression, exosomal microRNAs, signaling pathways, therapeutic mechanisms

## Abstract

Depression is a prevalent psychological disorder frequently associated with neuroinflammation, and microRNAs (miRNAs) have been implicated in its pathogenesis. Acupuncture, widely used in East Asia, has shown efficacy in various neuropsychiatric conditions; however, its miRNA‐related mechanisms remain unclear. Using an LPS‐induced depression model, we examined acupuncture's effects on depression‐like behaviors and circulating exosomal miRNAs in serum. Microarray data revealed multiple miRNAs significantly altered by LPS or acupuncture, and subsequent bioinformatics analyses (TargetScan, KEGG, and Gene Ontology) identified pathways related to neuroinflammation, synaptic signaling, and circadian regulation. Constructing a miRNA–target gene network further suggested that acupuncture might modulate miRNA expression to alleviate depressive symptoms. These findings not only support the therapeutic potential of acupuncture but also propose candidate exosomal miRNAs as novel biomarkers or diagnostic tools for the LPS‐induced depression model. Overall, this study provides insights into the anti‐inflammatory actions of acupuncture in depression through miRNA‐mediated gene regulation.

## 1 BACKGROUND

Depression is characterized by experiencing a wide spectrum of symptoms including depressed mood, cognitive dysfunction, fatigue, feeling of helplessness, and suicidal thought in serious cases (Maletic et al., 2007). Despite the high prevalence of depression among the global population, there are limited treatment methods being used in depression patients (Borroto‐Escuela et al., 2021; Jacobsen et al., 2012). Antidepressant, one of the most common treatments for depression, has been frequently used among the depressive population, and yet its efficiency in long‐term use is often questioned (Andrews et al., 2012). The causes of depression are complex and multifactorial. Imbalances in neurotransmitters like serotonin, dopamine, and norepinephrine are thought to play a central role in regulating mood, and many antidepressants target these systems. However, this mechanism does not fully explain treatment‐resistant depression, suggesting the involvement of other factors (Moncrieff et al., 2023). Additionally, reduced levels of brain‐derived neurotrophic factor (BDNF) impair neuroplasticity and lead to neuronal atrophy, which is linked to cognitive and emotional dysfunction in depression (Duman & Li, 2012).

Besides, growing evidence presents that the pathogenesis and progression of depressive disorder correlate with neuroinflammation (Troubat et al., 2021). Increased levels of proinflammatory cytokines in patients and animal models suggest that inflammation disrupts brain function, affecting neurotransmission, neurogenesis, and synaptic connectivity, which may worsen depressive symptoms (Afridi & Suk, 2021).

Exosomal miRNAs are detected in various body fluids including blood, urine, and saliva. miRNAs packed in exosomes can gain stability by avoiding degradation and contribute to various biological processes (Nik Mohamed Kamal & Shahidan, 2019). The role of circulating exosomal miRNA in neurological disorders has been gaining attention due to the well‐known post‐transcriptional function of miRNA and the ability to cross the blood–brain barrier (BBB) (McNeill & Van Vactor, 2012; Yu et al., 2021). Meanwhile, studies have found several miRNAs that are presumably related to the regulation of microglia‐mediated inflammatory response and neuronal differentiation (Guo et al., 2019; Zhao et al., 2021). miRNA studies suggested its contribution role in neuroinflammatory regulation and neuropathology in a wide range of physiological functions and cellular machinery (Gaudet et al., 2018).

Acupuncture treatment is widely recognized and practiced in East Asia, and its efficacies in many diseases have been gaining attention (Kawakita & Okada, 2014). Acupuncture at ST36 (Stomach meridian 36, Zusanli), one of the key acupoints in Traditional Chinese Medicine, has been shown to regulate neuroinflammation through mechanisms such as vagus nerve‐mediated cholinergic anti‐inflammatory responses and modulation of the TLR4/NF‐κB signaling pathway. Electroacupuncture (EA) at ST36 has been reported to reduce proinflammatory cytokines by enhancing cannabinoid receptor 2 (CB2R) expression and inhibiting calcium influx. Additionally, EA can activate the vagal–adrenal anti‐inflammatory axis, further supporting its protective effects (Liu et al., 2021). Given these established anti‐inflammatory and neuroprotective properties, ST36 was selected as the acupoint of interest in this study to investigate its effects on neuroinflammation and depression‐related pathology. Animal studies of acupuncture show its biological function and potential effects on diverse neuroinflammatory diseases (Zhang et al., 2023). Meanwhile, the association between acupuncture stimulation and miRNAs changes has been reported, and the results suggested the possibility of its potential effect on diverse diseases (Ko & Kim, 2019). Studies exploring the therapeutic effect of acupuncture via significant changes in miRNAs in various disease animal models such as hypertension (Wang et al., 2015) or stroke (Zheng et al., 2016) are growing.

To date, miRNAs have been actively studied and associated with brain diseases and the inflammatory response process; however, to our knowledge, no studies analyzed changes of miRNAs regarding neuroinflammation via acupuncture treatment in a mice model. Thus, in this study, we analyzed differentially expressed miRNAs in the serum from the LPS‐induced depression mice model and further investigated the interaction of the expression changes in serum miRNAs with the effect of acupuncture in terms of neuroinflammation.

## 2 MATERIALS AND METHODS

### 2.1 Animals and experimental design

**FIGURE 1:**
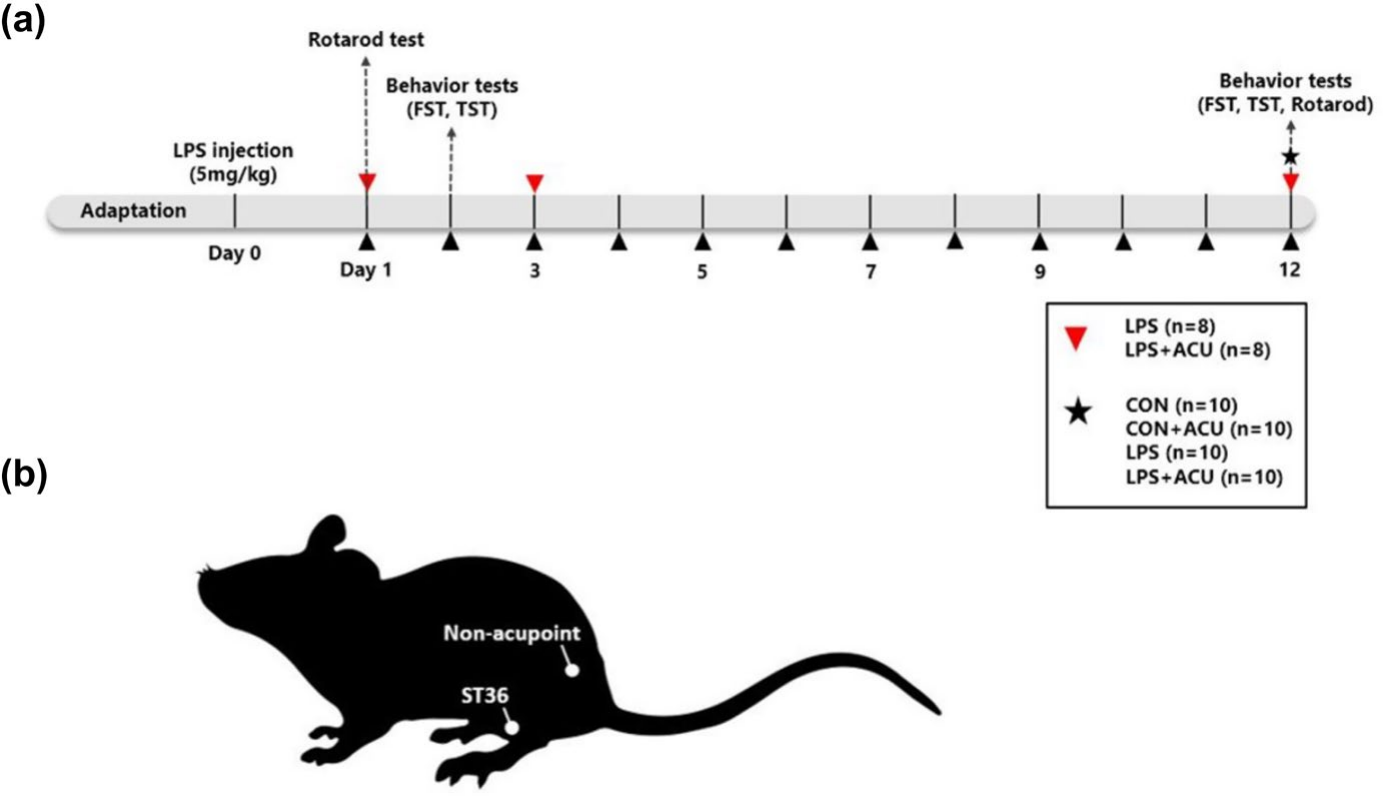
Timeline of the experiment and location of the acupoint. (a) Schematic diagram of the experimental timeline. The solid red arrowhead represents blood sampling. The black arrow heads indicate acupuncture treatments which performed daily for 12 days. The black dashed lines represent the day of conducting behavior tests. (b) Location of the acupoint (ST36) is shown in the diagram.

Eight‐week‐old male C57BL/6 mice (Orient‐Bio, Republic of Korea) were housed at 22°C ± 2°C, a humidity of 55 ± 5%, and a 12/12‐h dark/light cycle. Animals were given food (Cat. No. SFR+ 40 RMM; Woosung‐Bio, Republic of Korea) and water ad libitum. All experiments were performed according to the guidelines approved by the Dongguk University Animal Care Committee (DGU‐IACUC‐2021‐055‐1). After a week of adaptation, mice were randomly assigned to one of the following groups for behavior assessments: (1) control (CON); *n* = 8, (2) control + acupuncture at ST36 (CON+ST36); *n* = 8, (3) LPS; *n* = 9, and (4) LPS + acupuncture at ST36 (LPS + ST36); *n* = 10. LPS (5 mg/kg of body weight, Escherichia coli, 0111:B4, Sigma‐Aldrich, USA) was administered by i.p. injection to the mice, and an equal volume of saline was given to the control mice by i.p. injection. Acupuncture treatments were given 24 h after the LPS injection, and experiments proceeded according to the experiment schedule (Figure 1a).

### 2.2 Acupuncture treatment

Acupuncture treatment started 24 h after the LPS injection and was performed once daily for 12 consecutive days. Mice in acupuncture treatment groups (LPS + ST36 and CON+ST36) received acupuncture treatment at ST36, which is located on the tibialis anterior muscle (Figure 1b). During the acupuncture treatment, mice were slightly immobilized; then acupuncture needles (0.18 mm in diameter and 8 mm in length; DongBang Acupuncture Inc., Boryung, Korea) were inserted bilaterally to a depth of 3 mm and rotated bidirectionally for 30 s at a rate of two spins per second. The mice in control groups were also immobilized for the same amount of time to equalize the amount of stress.

### 2.3 Behavior test 1: Tail suspension test (TST)

Mice were individually suspended by securely attaching adhesive tape to the distal end of their tail, 30 cm above the floor, on a rod. Each animal was suspended for total of 6 min, and all test sessions were video recorded. Mice were considered immobile when they hung completely motionless. Each session lasted for a total of 6 min, and two observers, who were not informed about the treatments, recorded immobility time during the last 4 min of each test session. To minimize the influence of prior testing on subsequent behavior, the TST was performed first, followed by the FST after a 3‐h interval.

### 2.4 Behavior test 2: Forced swimming test (FST)

Mice were individually placed into transparent acrylic cylinders (20 cm diameter, 50 cm length) filled with 24 ± 1°C water to a 30 cm depth so that the mice could not touch the bottom with their tails or feet (Yankelevitch‐Yahav et al., 2015). The time recording started as soon as the animal was placed in the water, and the duration of each session was 6 min. The entire FST sessions were video recorded for later analysis of total immobile time. Immobility time was regarded as the mouse floating in the water without struggling. After testing, the mice were towel‐dried and placed under a heat lamp for 10 min before returning to their home cage. Two observers, who were not aware of the treatments, scored the immobility time of the last 4 min of each session.

### 2.5 Blood sample preparation and exosome isolation

On day 2, blood samples were collected via cardiac puncture from all the mice included in the experiments to obtain an adequate volume of serum for downstream analysis. Blood was left at room temperature for 20–30 min to clot; then serum was isolated by centrifugation at 3000 rpm for 15 min and stored at −80°C for further analysis.

Exosomes from 200 μL mouse serum were isolated by ExoQuick‐TC (Cat. No. EXOTC50A‐1, System Biosciences Inc., CA, USA) according to the manufacturer's instructions. In brief, the ExoQuick‐TC/serum mixture was incubated at 4°C for 30 min, then centrifuged at 13,000 rpm for 2 min. The supernatant was removed, and the pellet was used for subsequent experiments.

### 2.6 miRNA microanalysis

RNA purity and integrity were evaluated using the ND‐1000 Spectrophotometer (NanoDrop, ThermoScientific, USA) and Agilent 2100 Bioanalyzer (Agilent Technologies, USA). The Affymetrix GeneChip miRNA 4.0 array (Cat. No. 902411, Affymetrix, USA) was used according to the manufacturer's protocol. RNA samples (10 ng) were labeled with the FlashTag™ Biotin RNA Labeling Kit (Cat. No. 901910, Genisphere, USA). The labeled RNA was quantified, fractionated, and hybridized to the miRNA array according to the instructions of the manufacturer. The labeled RNA was heated on a block heater at 100°C for 5 min, followed by 45°C for 5 min. RNA‐array hybridization was performed with agitation at 60 rpm for 16 h at 48°C on the Affymetrix GeneChip, and the chips were washed and stained using the Affymetrix Fluidics Station 450 (Affymetrix, USA). The chips were then scanned using the Affymetrix GCS3000 scanner (Affymetrix, USA). The values of the signal were analyzed using the Affymetrix GeneChip Command Console software (AGCC, version 4.0.0; Affymetrix, USA).

### 2.7 miRNA statistical analysis and visualization

Raw data extraction was performed automatically according to the Affymetrix data extraction protocol using the AGCC software. After the CEL files were imported, the miRNA level RMA + DABG algorithm was applied using the Affymetrix® Power Tools (APT) Software, and the results were exported. Array data were filtered using probe‐annotated species. Comparative analysis between the LPS and LPS + ST36 samples was performed using *p*‐value, transcriptional levels, and fold‐change values. *p*‐values were determined by unpaired Student's *t*‐test for assessing the significance of differences between LPS and LPS + ST36. The false discovery rate (FDR) was controlled by adjusting the *p*‐value using the Benjamini‐Hochberg algorithm. After adjusting, statistical significance was set to *p* < 0.05 and |log2FC| >1. Visualization of differentially expressed genes was conducted using R statistical language v.4.2.2 and displayed as a volcano plot and heatmap.

### 2.8 miRNA target gene prediction and KEGG pathway and gene ontology (GO) analysis

The target genes of differentially expressed miRNAs were searched by using TargetScan (https://www.targetscan.org/vert_80/) (McGeary et al., 2019) and miRDB (https://mirdb.org/) (Chen & Wang, 2020). Target genes with cumulative weighted context++ score <−0.3 (by TargetScan) were selected and used for further analysis. Kyoto Encyclopedia of Genes and Genomes (KEGG) pathway analysis and visualization were performed using ShinyGO 0.76.3 (http://bioinformatics.sdstate.edu/go/) (Ge et al., 2020). Gene Ontology (GO) enrichment analysis was also performed using ShinyGO 0.76.3 and visualized by SRplot (https://www.bioinformatics.com.cn/en) (Tang et al., 2023). The potential regulatory relationships between miRNAs and target genes were analyzed using Cytoscape software (Shannon et al., 2003).

### 2.9 Statistical analysis

Experimental results are presented as means ± standard deviation (SD) and data were analyzed by One‐way analysis of variance (ANOVA) followed by Bonferroni's post hoc multiple comparison test by using GraphPad Prism (GraphPad Software Inc., La Jolla, CA, USA). *p*‐values less than 0.05 were considered statistically significant.

## 3 RESULTS

### 3.1 Effect of acupuncture on depression‐like behaviors in LPS‐administered mice

**FIGURE 2:**
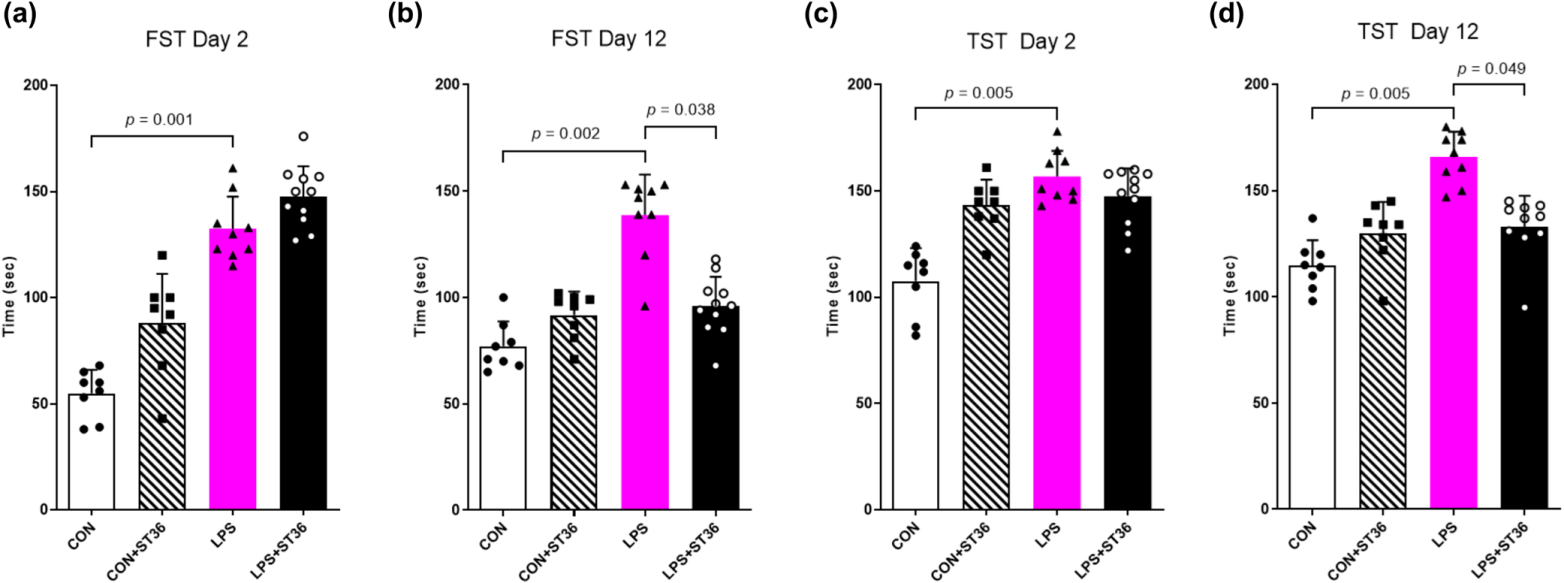
Behavior changes of LPS‐induced depression mouse model. Depressive‐like behaviors in mice were measured in the (a,b) forced swimming test and (c,d) tail suspension test. *p*‐values are displayed above the compared bars in each graph.

To examine LPS‐induced depressive behaviors in mice, FST was conducted (Figure 2a,b) at two different time points. On day 2 (Figure 2a), compared to the control group, significantly increased immobility time was observed in LPS‐injected mice, while there was no significantly different immobility time in LPS + ST36 group compared to LPS group. On day 12 (Figure 2b), compared to the control group, mice in the LPS group still showed significantly longer immobility time. Interestingly, after 12 days of acupuncture, a significant decrease in total immobile time was observed in LPS + ST36 group compared to LPS group. In the tail suspension test (Figure 2c,d), depressive behavior was evaluated by measuring the time during which mice remain immobile as they are being suspended by the tail. On day 2 (Figure 2c), there was increased time of immobility in the LPS group compared to the control group. The significant increase of immobility remained after 12 days of acupuncture treatment in the LPS group compared to the control group (Figure 2d). In contrast, the LPS + ST36 group showed a significant decrease in immobility time compared to the LPS group. Yet, immobility time in the CON + ST36 group was not significantly different from the CON group, which showed no treatment effect of acupuncture in control mice. These results suggest that acupuncture might exhibit a depression‐like behavior improvement effect in the mice model.

### 3.2 Analyses of differentially expressed exosomal miRNAs from LPS‐induced depressive mice serum

A variety of molecules, including enzymes, functional proteins, mRNAs, and miRNAs, are transported by exosomes, as they are protected by a lipid bilayer. Among the molecules, miRNAs are known to be the most numerous cargo molecules in the exosome, which are involved in various biological functions, including regulation of gene expression and post‐transcription (Zheng et al., 2020). Here, we analyzed profiles of miRNAs obtained from microanalysis and further identified potential target miRNAs associated with the inflammatory condition and acupuncture treatment.

**FIGURE 3:**
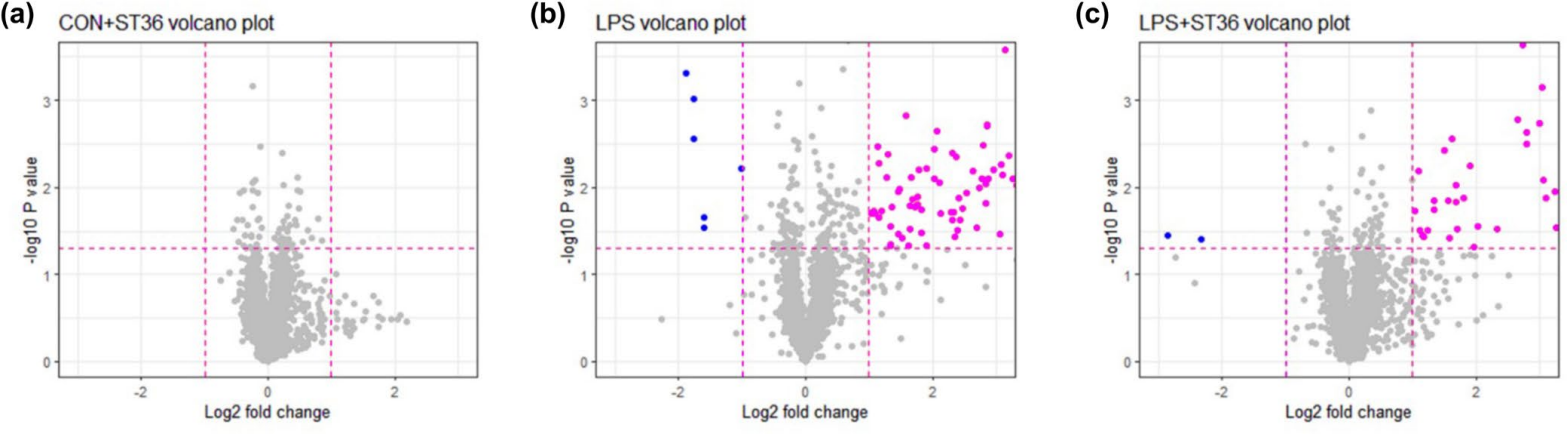
Volcano plot of differential miRNA expression. Volcano plots display (a) miRNA expression of CON+ST36 group compared to CON group, (b) miRNA expression of LPS group compared to CON group, and (c) miRNA expression on LPS+ ST36 group compared to CON group. Y‐axis represents the −log10 (*t*‐test, *p*‐value); X‐axis represents the log2 ratio of each miRNA. The magenta and blue color indicates the significantly up‐regulated miRNAs and significantly down‐regulated miRNAs, respectively.

The expression profile of exosomal miRNAs from mouse serum samples, which were collected 48 h after LPS injection, was analyzed. The volcano plots show differentially expressed miRNAs in exosome from serum of mice in CON+ST36 group (Figure 3a), LPS group (Figure 3b), and LPS + ST36 (Figure 3c). MiRNAs, which are *p* < 0.05 and |log2FC| >1, were considered as significantly up‐regulated and then selected for further analysis in this study. There were 75 significantly up‐regulated miRNAs in LPS group and 40 significantly up‐regulated miRNAs in LPS + ST36 group, while no miRNA significantly changed expression in CON+ST36 group.

**FIGURE 4:**
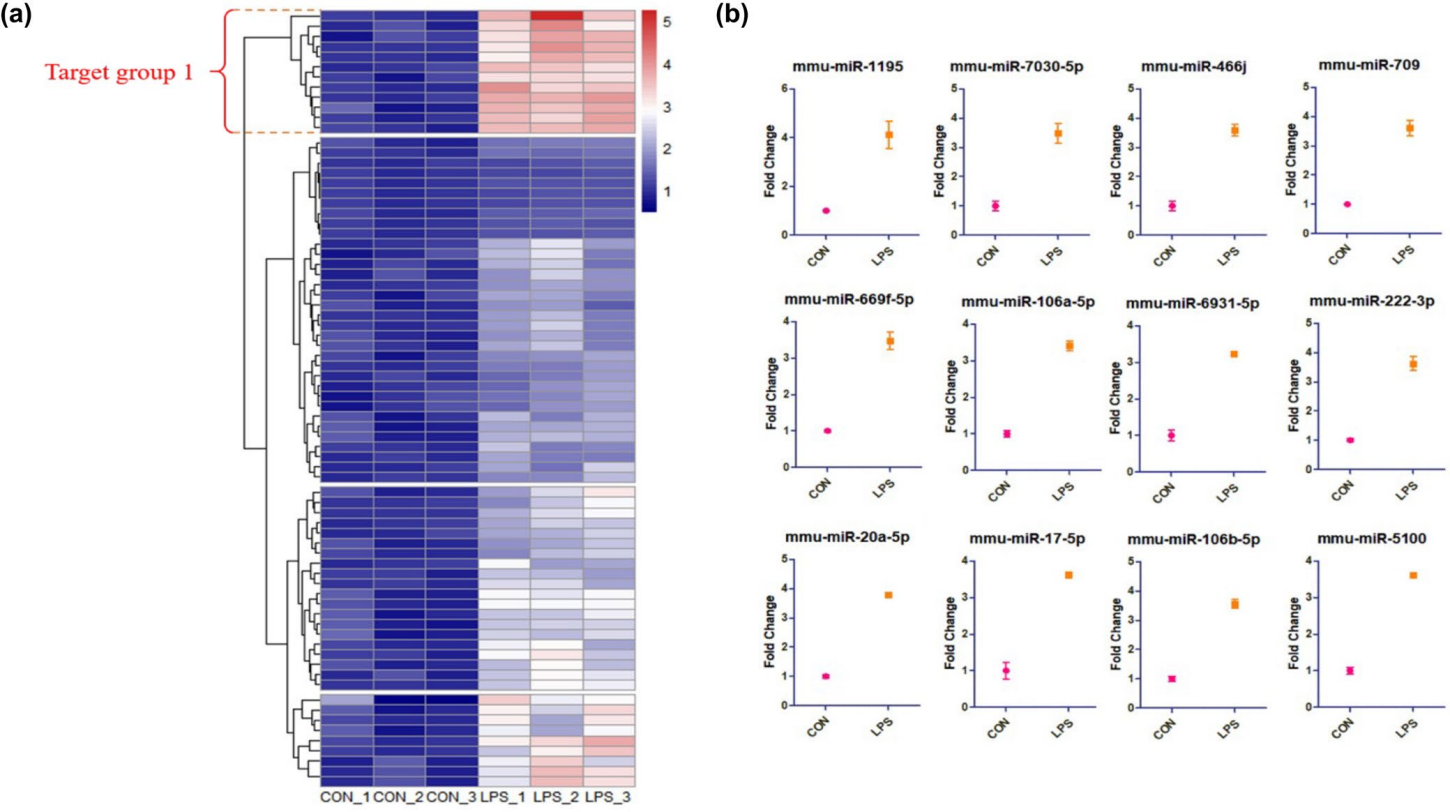
Heatmap with up‐regulated miRNAs in LPS group, compared with CON group. (a) Heatmap displays distribution of different exosomal miRNA expressions changes in serum of LPS group mice (*n* = 3) compared to mice in CON group (*n* = 3). The horizontal axis represents experimental groups, and the vertical axis shows miRNAs. Blue indicates lower level of miRNAs and the smaller the value, darker the color. Red indicates higher level of miRNAs and the larger the value, the darker the color. (b) Different miRNA expressions of Target group 1. Y‐axis represents Fold Change.

**FIGURE 5:**
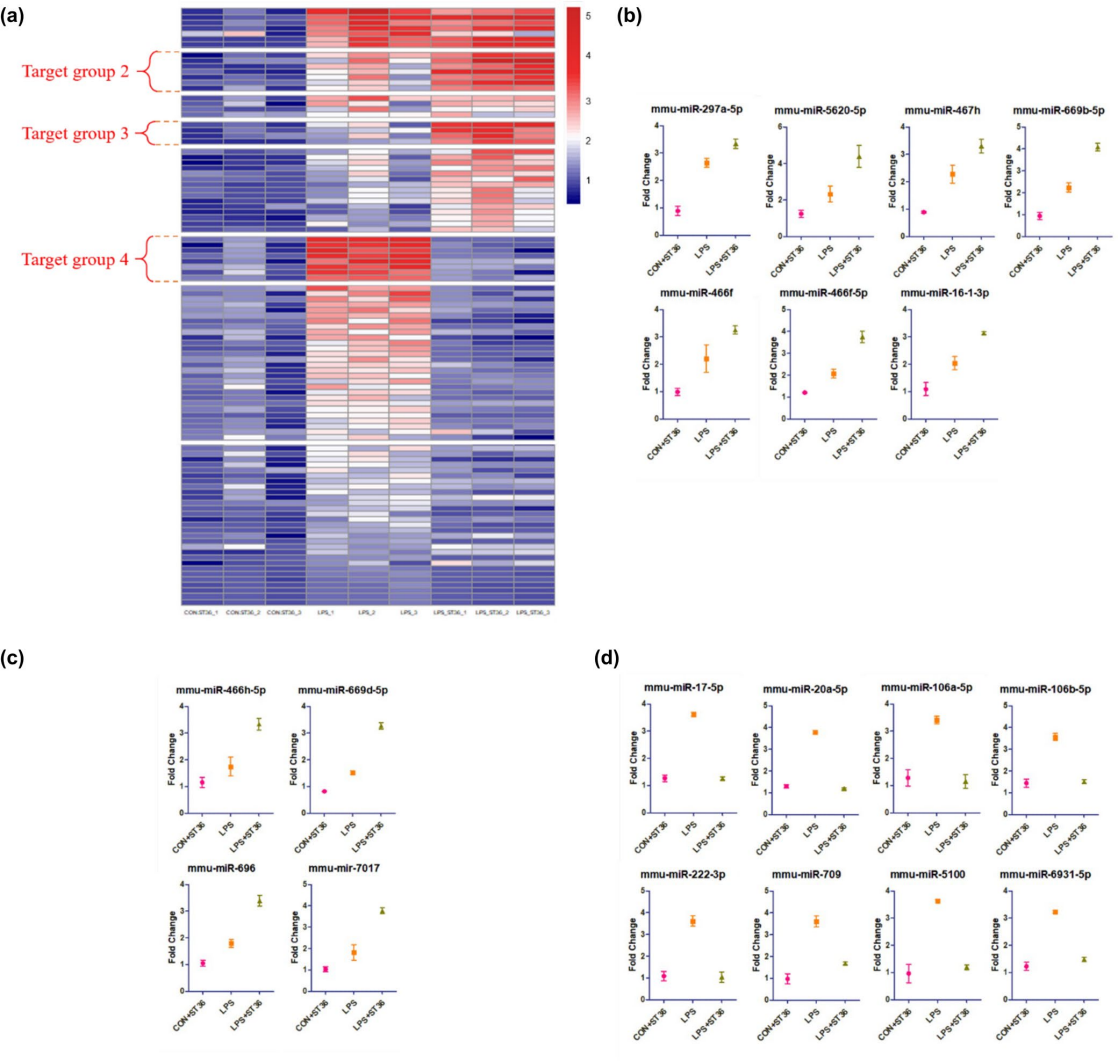
Heatmap with up‐regulated miRNAs in LPS + ST36 group, compared with CON+ST36 and LPS group. (a) Heatmap displays distribution of different exosomal miRNA expressions changes in serum of CON+ST36 group mice (*n* = 3), LPS group mice (*n* = 3), and LPS + ST36 group mice (*n* = 3). The horizontal axis represents experimental groups, and the vertical axis shows miRNAs. Blue indicates lower level of miRNAs and the smaller the value, darker the color. Red indicates higher level of miRNAs and the larger the value, the darker the color. Each plot on (b–d) display differential miRNA expressions of Target group 2, Target group 3, and Target group 4, respectively.

Based on the volcano plot analysis, the profile of significantly up‐regulated miRNAs in the LPS group and LPS + ST36 group was used to further examine the different expressions between the CON + ST36 group, LPS group, and LPS + ST36 group as compared to CON group values. We were able to set four groups of target miRNAs. Target group 1 includes miRNAs with significantly high expression in the LPS group compared to the CON group. Target group 2 includes miRNAs with significantly high expression in both the LPS group and LPS + ST36 group, with higher expression in the LPS + ST36 group than in the LPS group (Figure 4a). Target group 3 includes miRNAs with significantly high expression in only the LPS + ST36 group. Target group 4 includes miRNAs with significantly high expression in only the LPS group (Figure 5a). In detail, expressions of miRNAs mmu‐miR‐1195, mmu‐miR‐7030‐5p, mmu‐miR‐466j, mmu‐miR‐709, mmu‐miR‐669f‐5p, mmu‐miR‐106a‐5p, mmu‐miR‐6931‐5p, mmu‐miR‐222‐3p, mmu‐miR‐20a‐5p, mmu‐miR‐17‐5p, mmu‐miR‐106b‐5p, and mmu‐miR‐5100, a total of 12 miRNAs, showed significantly higher expression in the LPS group and were set as target group 1 (Figure 4b). A total of 7 miRNAs with significantly high expression in both the LPS group and LPS + ST36 group, with higher expression in the LPS + ST36 group than in the LPS group: mmu‐miR‐297a‐5p, mmu‐miR‐5620‐5p, mmu‐miR‐467 h, mmu‐miR‐669b‐5p, mmu‐miR‐466f, mmu‐miR‐466f‐5p, and mmu‐miR‐16‐1‐3p were identified and set as target group 2 (Figure 5b). Mmu‐miR‐7017, mmu‐miR‐696, mmu‐miR‐466 h‐5p, and mmu‐miR‐669d‐5p, a total of 4 miRNAs, showed significantly higher expression in only LPS + ST36 group and set as target group 3 (Figure 5c). Finally, target group 4 was set by a total of 8 miRNAs with significantly high expression in only the LPS group: mmu‐miR‐20a‐5p, mmu‐miR‐5100, mmu‐miR‐222‐3p, mmu‐miR‐17‐5p, mmu‐miR‐709, mmu‐miR‐106b‐5p, mmu‐miR‐106a‐5p, and mmu‐miR‐6931‐5p (Figure 5d).

### 3.3 KEGG pathway analysis for miRNAs signature target genes

**FIGURE 6:**
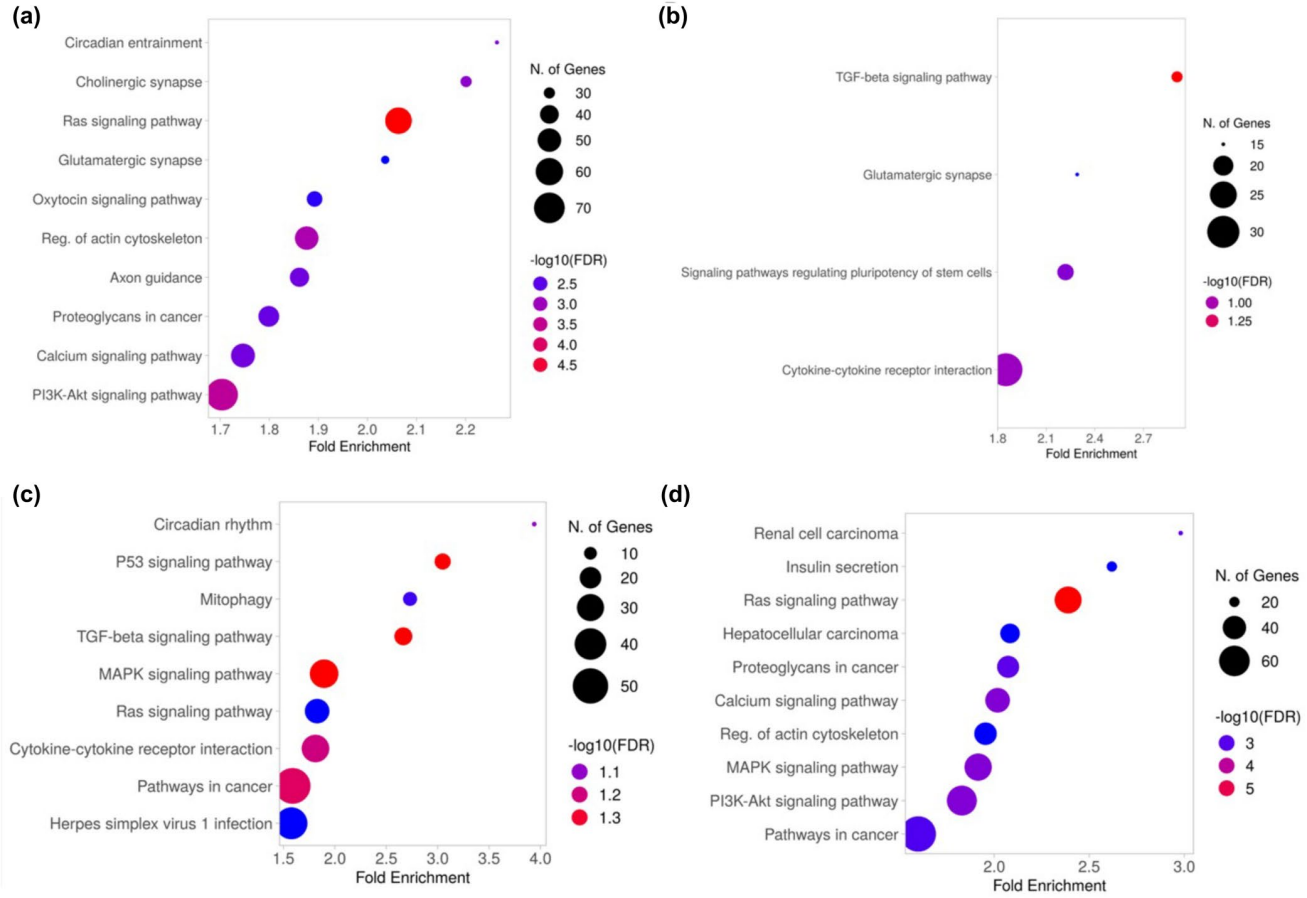
KEGG analysis on differentially expressed target miRNAs. KEGG pathway analysis results for differentially expressed target miRNAs on each target group were performed by bubble. Y‐axis represents the KEGG pathways, X‐axis represents the fold enrichment for each pathway, and the size and color of each bubble represent the number of genes and the –log10(FDR). Each plot of (a–d) are results of target groups 1, 2, 3, and 4, respectively.

To comprehensively examine the biological relevance between significantly up‐regulated miRNAs in each target group and their predicted target genes, KEGG pathway analysis for the miRNA‐target pairs was performed. Potential target pathways for the miRNAs in target group 1 include circadian entrainment, cholinergic synapse, and Ras signaling pathway. For the miRNAs in target group 2, TGF‐beta signaling pathway, glutamatergic synapse, and signaling pathways regulating pluripotency of stem cells were found as target pathways. In target group 3, Circadian rhythm, p53 signaling pathway, and mitophagy were found as target pathways. Renal cell carcinoma, insulin secretion, and Ras signaling pathway were found as target pathways for the miRNAs in target group 4 (Figure 6).

### 3.4 Gene ontology (GO) analysis for miRNAs signature target genes

**FIGURE 7:**
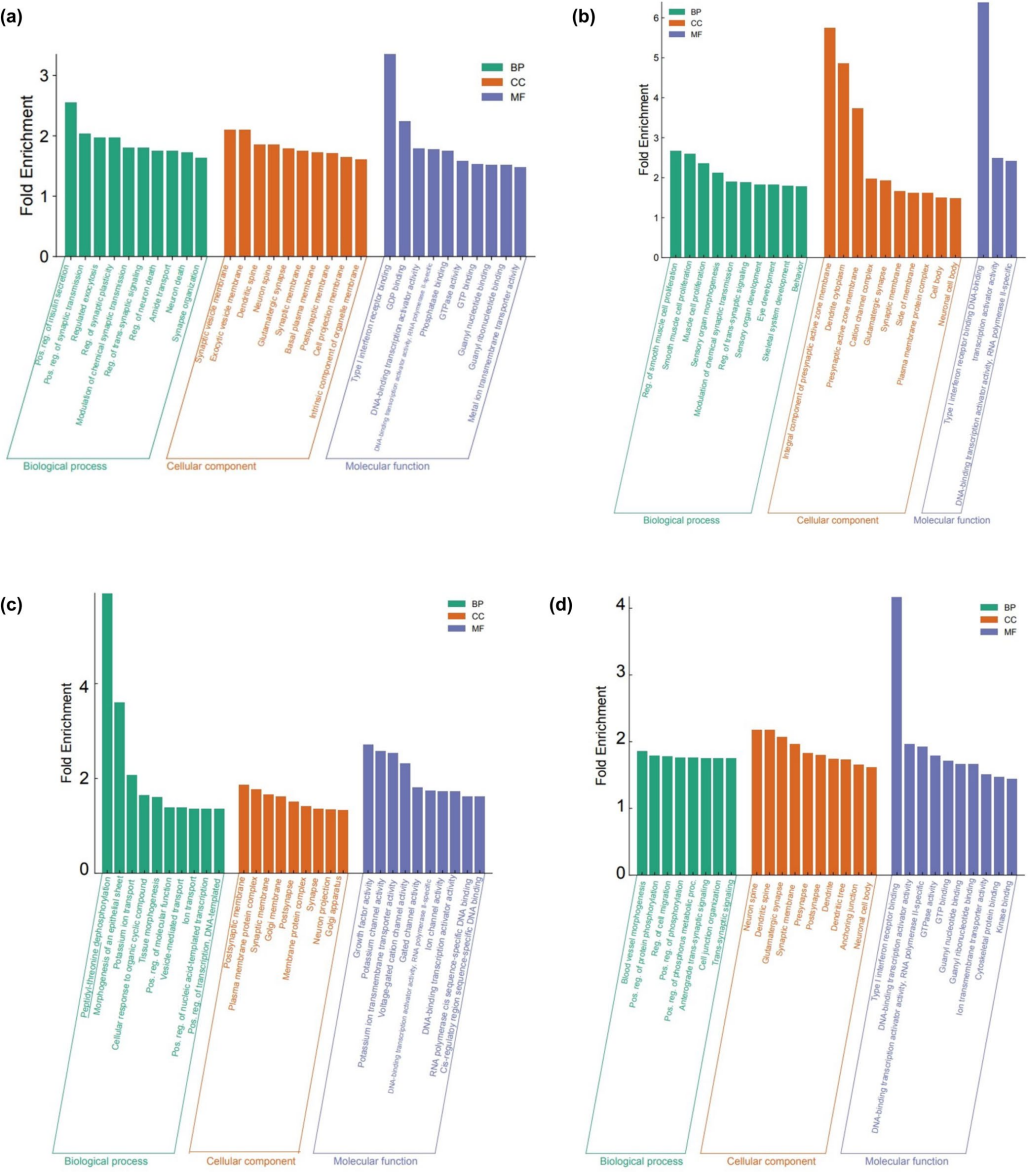
GO analysis on differentially expressed target miRNAs. GO enrichment analysis results for predicted target genes of (a) target group 1, (b) target group 2, (c) target group 3, and (d) target group 4. These were selected by FDR, sorted by fold enrichment, and visualized by bar charts. The green, red, and blue color of X‐axis respectively indicates the top 10 GO terms for Biological Process, Molecular Function, Cellular Component. Y‐axis represents Fold Enrichment.

To make a better understanding of the functions of predicted targets for miRNAs, Gene Ontology (GO) analyses were performed on each target group that are associated with biological process (BP), cellular components (CC), and molecular function (MF). Up to the top 10 GO terms in each of the three major categories were selected by FDR and sorted by fold enrichment. For target group 1 miRNAs, the most enriched GO terms included positive regulation of synaptic transmission in BP, synaptic vesicle membrane in CC, and type I interferon receptor binding in MF. The most enriched GO terms in target group 2 miRNAs were smooth muscle cell proliferation, integral component of presynaptic active zone membrane, and type I interferon receptor binding in BP, CC, and MF respectively. Morphogenesis of an epithelial sheet in BP, postsynaptic membrane in CC, and growth factor activity in MF were found as the most enriched GO terms in target group 3. Positive regulation of protein phosphorylation in BP, neuron spine in CC, and type I interferon receptor binding in MF were the most enriched GO terms in target group 4 (Figure 7).

### 3.5 Construction of a regulatory network for miRNAs signature target genes

**FIGURE 8:**
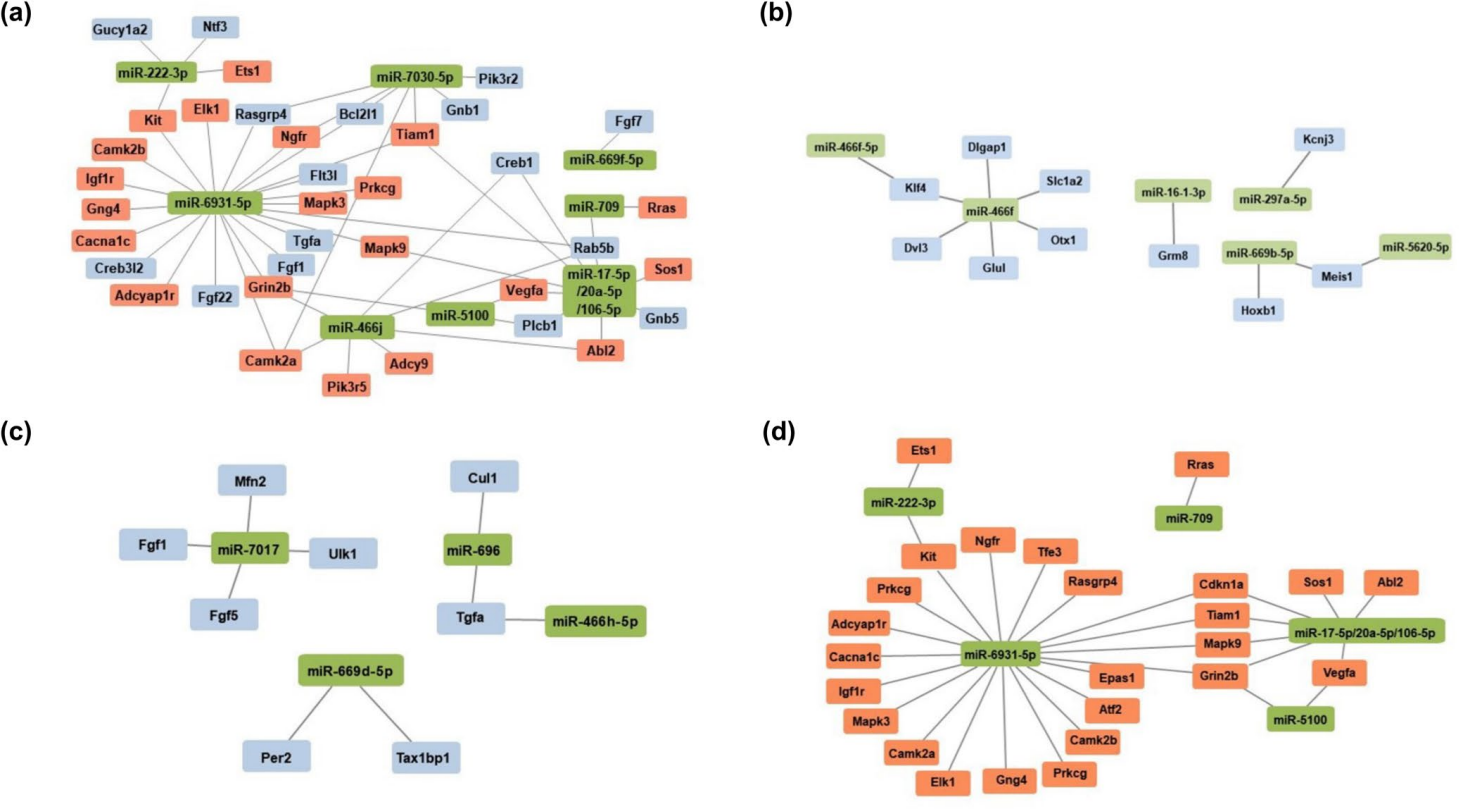
Visualization of miRNAs and their associated target genes network on each target group. The red rectangles indicate the target genes predicted to be associated with inflammation, the blue rectangles indicate the target genes which were supposed to be involved in the compensation mechanism. The green rectangles indicate the target miRNAs of each group. Each of (a–d) represents target group 1, 2, 3, and 4, respectively.

To identify differentially expressed miRNAs on each target group and investigate the biological processes affected by these miRNAs by analyzing their predicted target genes, the pathway analysis of miRNA‐target gene interaction was conducted using Cytoscape software. In each target group, we classified target genes into those related to inflammation and those related to compensation or therapeutic mechanisms. As a result, we predicted that target genes in target group 1, which were up‐regulated in the LPS group compared to the CON group, were related to inflammatory effects or the body's own compensation mechanism by LPS. Target genes of target group 2, which included up‐regulated miRNAs in both the LPS group and the LPS + ST36 group with higher levels in the LPS + ST36 group, were predicted as related to compensation mechanisms. We considered target genes of target group 3, which were up‐regulated only in the LPS + ST36 group, as genes associated with the therapeutic mechanism. Lastly, we considered the target gene of target group 4, which was up‐regulated only in the LPS group, as the gene associated with inflammation (Figure 8).

## 4 DISCUSSION

MicroRNA (miRNA), a small non‐coding RNA, has gained attention for its potential gene regulatory role in various diseases (Chipman & Pasquinelli, 2019; O'Brien et al., 2018). Among various non‐coding RNAs, including snoRNA, siRNA, and long non‐coding RNA, miRNA is mostly studied and suggested as a significant regulator of neuronal activity and brain functions (Brennan & Henshall, 2020; Thomas et al., 2018). Circulating miRNAs are packed and transported in vesicles, and mostly encapsulated in exosomes as being protected by a rigid bilayer from degradation (Gurung et al., 2021). Increasing evidence suggests that exosomal miRNAs are involved in the pathophysiology of various neurologic disorders (Xia et al., 2019). Moreover, due to the characteristics of exosomes transporting various molecules and active involvement in cellular communications, as well as the unique post‐transcriptional aspect of miRNAs (Colombo et al., 2014), there has been markedly increasing interest in the role of exosomal miRNA and the application of exosomal miRNAs as potential biomarkers and therapeutic agents of a wide range of diseases (Kosaka et al., 2010).

Given the knowledge of the unique gene regulatory role of exosomal miRNAs, we examined differentially expressed exosomal miRNAs that were isolated from LPS‐induced neuroinflammation mice serum. We analyzed profiles based on the volcano plot of miRNA expression and identified up‐regulated miRNAs in the LPS group and the LPS + ST36 group to further investigate the different expression between the CON + ST36 group, the LPS group, and the LPS + ST36 group by comparison with CON group values. The analysis of miRNA profiles was further analyzed and categorized into four target groups. Target group 1 contains miRNAs with significantly high expression in the LPS group in comparison with the CON group. Target group 2 contains miRNAs with considerably high expression in both the LPS group and the LPS + ST36 group, with higher expression in the LPS + ST36 group against the LPS group. Target groups 3 and 4 contain miRNAs that had remarkably high expression in the single groups of the LPS + ST36 group and the LPS group, respectively.

To understand the role of multiple miRNAs and their biological function, enrichment analysis was conducted on differently expressed targeted miRNAs using KEGG analysis and GO analysis. We further characterized multiple miRNAs and their target genes by a regulatory network, which is visualized in Figure 8.

Interestingly, we found several miRNAs that might be able to play a role as a biomarker in neuroinflammation‐induced depression and the anti‐inflammatory effect of acupuncture. Increased levels of multiple miRNAs involved in the target group 4, including mmu‐miR‐20a‐5p, mmu‐miR‐5100, mmu‐miR‐222‐3p, mmu‐miR‐17‐5p, mmu‐miR‐709, mmu‐miR‐106b‐5p, mmu‐miR‐106a‐5p, and mmu‐miR‐6931‐5p, showed high levels in the LPS group but low levels in the LPS + ST36 group. The different levels of miRNAs between the LPS and LPS + ST36 groups might suggest the potential role of the anti‐inflammatory effect that occurred due to acupuncture stimulation. In this respect, the anti‐inflammatory effect of acupuncture treatment seems to be associated with the miRNAs regulatory network.

MiRNAs involved in the target group 3, such as mmu‐miR‐7017, mmu‐miR‐696, mmu‐miR‐466 h‐5p, and mmu‐miR‐669d‐5p, are pretended to be related to therapeutic mechanisms because these miRNAs increased in the acupuncture treatment group of LPS + ST36. Several studies of downstream genes, including Mfn2 (Li et al., 2022), Fgf1 (Dhlamini et al., 2022), Per2 (Russell et al., 2021), and Tax1bp1 (Wu et al., 2023) of target group 3 were evidenced to be related to the anti‐inflammatory effect in multiple disease models. Altogether, the correlation of circulating miRNAs and their target genes in group 3 suggests the biological function of anti‐inflammatory.

Notably, the change of miRNAs in target group 2, including mmu‐miR‐297a‐5p, mmu‐miR‐5620‐5p, mmu‐miR‐467 h, mmu‐miR‐669b‐5p, mmu‐miR‐466f, mmu‐miR‐466f‐5p, and mmu‐miR‐16‐1‐3p, was significant. These miRNAs both increased in the group of LPS and LPS + ST36, but the LPS + ST36 group showed higher expression against the LPS group. Among downstream genes of target group 2, two genes were reported to have both sides of inflammation. Klf4 not only showed vascular protection (Zhang et al., 2020) on ischemic stroke but also activation of inflammatory injury (Chen et al., 2022). Dvl3 was both related to inflammation (Zhang et al., 2018) and anti‐inflammatory effect (Yang et al., 2020). Still, it is unclear why miRNAs in the LPS + ST36 group were increased higher than in the LPS group; we can speculate that acupuncture possibly activated a different side of the mechanism of these miRNAs in inflammation. These detailed mechanisms in inflammation are required to be clarified by further studies.

In this report, we found the change of miRNAs in the serum from a neuroinflammation‐induced depression mice model treated with acupuncture. The results indicate that acupuncture stimulation enhances the possibility of serum miRNAs to play as a biomarker in neuroinflammation‐related disease. The results of our analysis suggested that the anti‐inflammatory effect of acupuncture treatment is via the miRNAs regulatory network. Still, the accurate process of how acupuncture regulates the changes of serum miRNAs needs to be kept studied. We hope the present study might be helpful for further acupuncture studies to explore its biological function associated with miRNAs.

## AUTHOR CONTRIBUTIONS

Conceptualization, J.H.K.; investigation, J.H.K. and K.Y.C.; writing—original draft preparation, J.H.K. and K.Y.C.; writing—review and editing, S.‐N.K.; supervision, S.‐N.K.

## FUNDING INFORMATION

This work was supported by the National Research Foundation of Korea funded by the Korean government (MSIT) (NRF‐2020R1C1C1004107).

## CONFLICT OF INTEREST STATEMENT

The authors declare that they have no competing interests.

## ETHICS STATEMENT

All experiments were performed according to the guidelines approved by the Dongguk University Animal Care Committee (DGU‐IACUC‐2021‐055‐1).

## Data Availability

The datasets used and/or analyzed during the current study are available from the corresponding author on reasonable request.
